# Fast and accurate representations of stochastic ion channel fluctuations

**DOI:** 10.1186/1471-2202-16-S1-P258

**Published:** 2015-12-18

**Authors:** David F Anderson, Bard Ermentrout, David D Friel, Roberto F Galán, Benjamin Lindner, Shusen Pu, Deena R Schmidt, Peter J Thomas

**Affiliations:** 1 Department of Mathematics, University of Wisconsin, Madison, WI 53706, USA; 2 Department of Mathematics, University of Pittsburgh, Pittsburgh, PA 15260, USA; 3 Department of Neurosciences, Case Western Reserve University, Cleveland, OH 44106, USA; 4 Bernstein Center for Computational Neuroscience, 10115 Berlin, Germany; 5 Department of Physics, Humboldt University, 12489 Berlin, Germany; 6 Department of Mathematics, Applied Mathematics and Statistics, Case Western Reserve University, Cleveland, OH 44106, USA; 7 Department of Mathematics and Statistics, University of Nevada, Reno, NV 89557, USA

## 

Random ion channel gating is an important source of noise at the single neuron level. For mesoscale ion channel population sizes, fast, accurate representation of channel noise fluctuations remains an important challenge. We highlight recent progress in three areas.

In [1] we present an exact stochastic simulation algorithm, which takes into account the time dependence of the voltage sensitive transitions due to rapid voltage changes during action potentials. The exact algorithm is similar to a widely used approximate stochastic simulation algorithm, in which transition rates are held fixed during the intervals between channel state transitions. We compare the algorithms and show that they are inequivalent in a strong sense, meaning that sample paths diverge when driven with identical Poisson processes, leading to different precise firing times. But the stationary histograms produced are practically indistinguishable for modest channel numbers (*circa *N>100), indicating weak equivalence.

For channel numbers in the hundreds or higher, numerical stochastic differential equations (SDE) algorithms based on the system size expansion can be significantly faster than simulations based on discrete Markov chain simulations, while retaining reasonable accuracy. For large networks, however, even SDE based simulations become costly, particularly as more complex channel gating schemes are introduced. Schmandt and Galán introduced a stochastic shielding approximation as a fast, accurate way of simulating stochastic ion channel kinetics [2]. In the SDE representation, each edge in the graph generates both a mean population flux between adjacent nodes, and a fluctuation about the mean. Only the fluctuations arising from edges connecting functionally distinct states directly affect fluctuations in the observed behavior of the cell. In [3] we analyze stochastic shielding both for the HH sodium and potassium gating models, and for an ensemble of random graphs. We derive a quantitative measure of edge importance related to the eigenvalue/eigenvector decomposition of the graph Laplacian matrix.

Channel noise makes a regularly spiking neuron a stochastic oscillator. The classical definition for the asymptotic phase of an oscillator breaks down when stochasticity is taken into account. Alternative definitions of the ``phase'' have been based on the mean first passage time property of a system of isochronal surfaces [4] and in terms of the spectral decomposition of a the adjoint Kolmogorov operator [5,6]. In the vanishing noise limit, the spectrally derived isochrons approach those of the underlying mean field system, if the latter has a finite period limit cycle.

Together, these results expand our analytic, numerical, and conceptual tools for understanding the effects of random ion channel gating in conductance based neural models.
